# Through the orbit and beyond: Current state and future perspectives in endoscopic orbital surgery on behalf of the EANS frontiers committee in orbital tumors and the EANS skull base section

**DOI:** 10.1016/j.bas.2023.102669

**Published:** 2023-08-28

**Authors:** C. Zoia, G. Mantovani, M. Müther, E. Suero Molina, A. Scerrati, P. De Bonis, J.F. Cornelius, P.H. Roche, M. Tatagiba, E. Jouanneau, R. Manet, H.W.S. Schroeder, L.M. Cavallo, E.M. Kasper, T.R. Meling, D. Mazzatenta, R.T. Daniel, M. Messerer, M. Visocchi, S. Froelich, M. Bruneau, G. Spena

**Affiliations:** a UOC Neurochirurgia, Ospedale Moriggia Pelascini, Gravedona e Uniti, Italy; b Neurosurgery Unit, Department of Translational Medicine, University of Ferrara, Ferrara, Italy; c Department of Neurosurgery, University Hospital of Münster, Münster, Germany; d Department of Neurosurgery, Medical Faculty, Heinrich-Heine-University, Düsseldorf, Germany; e Department of Neurosurgery, Aix-Marseille Université, Assistance Publique-Hôpitaux de Marseille, Hôpital Nord, Marseille, France; f Department of Neurosurgery, University Hospital Tübingen, Tübingen, Germany; g Department of Neurosurgery, Hôpital Neurologique Pierre Wertheimer, Lyon, France; h Department of Neurosurgery, University Medicine Greifswald, Germany; i Department of Neurosciences and Reproductive and Dental Sciences, Division of Neurosurgery, Federico II University of Naples, Policlinico Federico II University Hospital, Italy; j Department of Neurosurgery, Steward Medical Group, Brighton, USA; k Department of Neurosurgery, The National Hospital, Rigshospitalet, Copenhagen, Denmark; l Department of Neurosurgery, Neurological Sciences Institut IRCCS, Bologna, Italy; m Department of Neurosurgery, Department of Neuroscience, Centre Hospitalier Universitaire Vaudois, University Hospital, Lausanne, Switzerland; n Department of Neurosurgery, Institute of Neurosurgery Catholic University of Rome, Italy; o Department of Neurosurgery, Lariboisière Hospital, Université Paris Diderot, Paris, France; p Department of Neurosurgery, Universitair Ziekenhuis Brussel, Vrije Universiteit Brussel, Brussels, Belgium; q Neurosurgery Unit, IRCSS San Matteo Hospital, Pavia, Italy

**Keywords:** Orbital surgery, Endoscopy, Transorbital, Skull base, Neuroendoscopy

## Abstract

**Introduction:**

Orbital surgery has always been disputed among specialists, mainly neurosurgeons, otorhinolaryngologists, maxillofacial surgeons and ophthalmologists. The orbit is a borderland between intra- and extracranial compartments; Krönlein's lateral orbitotomy and the orbitozygomatic infratemporal approach are the historical milestones of modern orbital-cranial surgery.

**Research question:**

Since its first implementation, endoscopy has significantly impacted neurosurgery, changing perspectives and approaches to the skull base. Since its first application in 2009, transorbital endoscopic surgery opened the way for new surgical scenario, previously feasible only with extensive tissue dissection.

**Material and methods:**

A PRISMA based literature search was performed to select the most relevant papers on the topic.

**Results:**

Here, we provide a narrative review on the current state and future trends in endoscopic orbital surgery.

**Discussion and conclusion:**

This manuscript is a joint effort of the EANS frontiers committee in orbital tumors and the EANS skull base section.

## Highlights

• Orbital surgery is a frontier between different specialties: neurosurgeons, otorhinolaryngologists, maxillofacial surgeons, and ophthalmologists. Endoscopy opened a whole new scenario of surgical possibilities.
• Trans-Orbital surgery seems to provide useful access to different intracranial compartments, previously reachable only via an extensive tissue dissection, with good functional and oncological outcomes.
• Homogeneous nomenclature of approaches and anatomical landmarks, data consistency across studies, broad shared classifications and well-designed randomized clinical trials are strongly advocated.
• Next years will be crucial to precise indications, techniques, outcomes and complications of trans-orbital endoscopy through data obtained from high-level scientific studies.
• Specific and dedicated applications are needed for transorbital endoscopic surgery to incorporate this topic into the common neurosurgical knowledge of the new generations.

## 1 Introduction

The orbit, its boundaries and contents are a frontier between different specialties: neurosurgeons, otorhinolaryngologists, maxillofacial surgeons, and ophthalmologists. Historically, surgeons from different specialties have addressed the pathologies that cross the orbital borders, such as orbital-cranial or sinus tumors invading the orbit, differently; in a continuous “*clash of surgical titans*” (Houlihan et al., 2021a). From their point of view, neurosurgeons always approached the orbit as a target to treat pathologies reaching out or into the cranium or as a keyhole to reach deep-seated regions of the brain, thereby using the orbit as a looking glass through which new surgical scenarios and possibilities have emerged.

In the late 1800s, ophthalmologists initiated the quest for safer access to the orbit as an evolution from orbital exenteration, the only available procedure for retrobulbar tumors for centuries. Starting from the first anterior orbitotomy, performed by Hermann Knapp in 1874 (H. Knapp), another milestone was reached with the lateral orbitotomy in 1889, also known as “*Krönlein's operation* (Krönlein, 1889), that remained a gold standard for decades (Meling, 2019). Meanwhile, neurosurgeons were also trying to push the boundaries between intra- and extracranial pathologies (Houlihan et al., 2021a), an example being Charles Frazier proposing a supraorbital ridge craniotomy to approach lesions of the sella turcica in 1913 (Frazier, 1913). In 1941, Dandy published a book on orbital tumors (Dandy, 1941). He strongly advocated for transcranial approach for all optic nerve tumors, instead of a transorbital one, stating that “*The only safe attack is the transcranial one*” (Dandy, 1945).

The first one to use the orbit as an access corridor to the brain, was the Italian psychiatrist Amarro Fiamberti, credited for being the original descriptor of the transorbital prefrontal lobotomy in 1937 (Fiamberti, 1937), which later came to be widely known after the work on psychosurgery by Freeman and Watts (Freeman, 1948) around 1948.

**Fig. 1:**
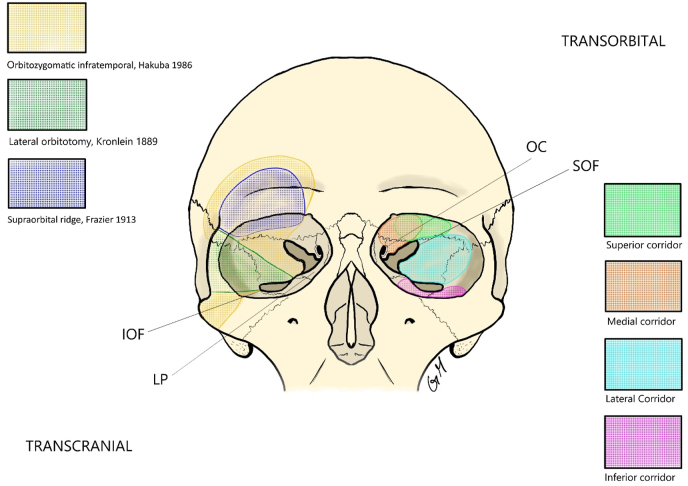
Principal approaches to the orbit. OC Optic Canal, IOF Inferior Orbital Fissure, LP Lamina Papyracea, SOF Superior Orbital Fissure.

With the evolution of microsurgery over the second half of the 20th century, skull base approaches began including principles of minimal tissue damage and optimal cosmesis. With this came the necessity to deal with the superior boundaries of the orbit to best treat anterior and middle cranial fossa pathologies. Thus, orbitotomy came to be incorporated into routine practices. In 1986, Hakuba Hakuba et al. (1986) described the orbitozygomatic infratemporal approach, perhaps the most crucial advancement since Krönlein's operation. Several modifications have been proposed to the classic orbitotomy techniques over the years, varying the degree of exposure (Abou-Al-Shaar et al., 2020) (Fig. 1).

The impact of endoscopy in neurosurgery has been tremendous. It has transformed transsphenoidal surgery and far beyond. Since its start in the late 20th century, it has been clear that endoscopy could provide new accesses to complex anatomical regions, including the orbit. By then, two parallel approaches have evolved to reach and cross the orbit endoscopically: the transorbital and transnasal approaches (Schwartz et al., 2022; Reisch e and Perneczky, 2005).

This narrative review is a combined effort of the EANS Skull base section and the orbital tumor task force of the EANS Frontiers section. We focus on the history of endoscopic approaches to the orbit, its current topics, and future directions from a neurosurgical point of view.

## 2 Material and methods

Literature was searched thoroughly to analyze the main articles concerning orbital surgery in accordance with the Preferred Reporting Items for Systematic Reviews and Meta-Analysis (PRISMA) statement.

The electronic databases PubMed (Medline), Cochrane Library, Ovid MEDLINE, and Scopus were searched using the following Medical Subject Headings (MeSH) and keywords: “transorbital”, “endoscopic”, “neuroendoscopic”, “approach” and “skull base”. MeSH and keywords search of each database was performed using the Boolean operators OR and AND, including randomized controlled trials (RCTs), cohort studies, case-control studies, case series, case reports, systematic and narrative reviews on human subjects. Only English article were included. No time limits were settled.

Papers were selected by the authors after abstract reading, based on their relevance to the topic of this narrative review. Citation tracking and references checking were performed, searching for other relevant papers.

## 3 Results

**Fig. 2:**
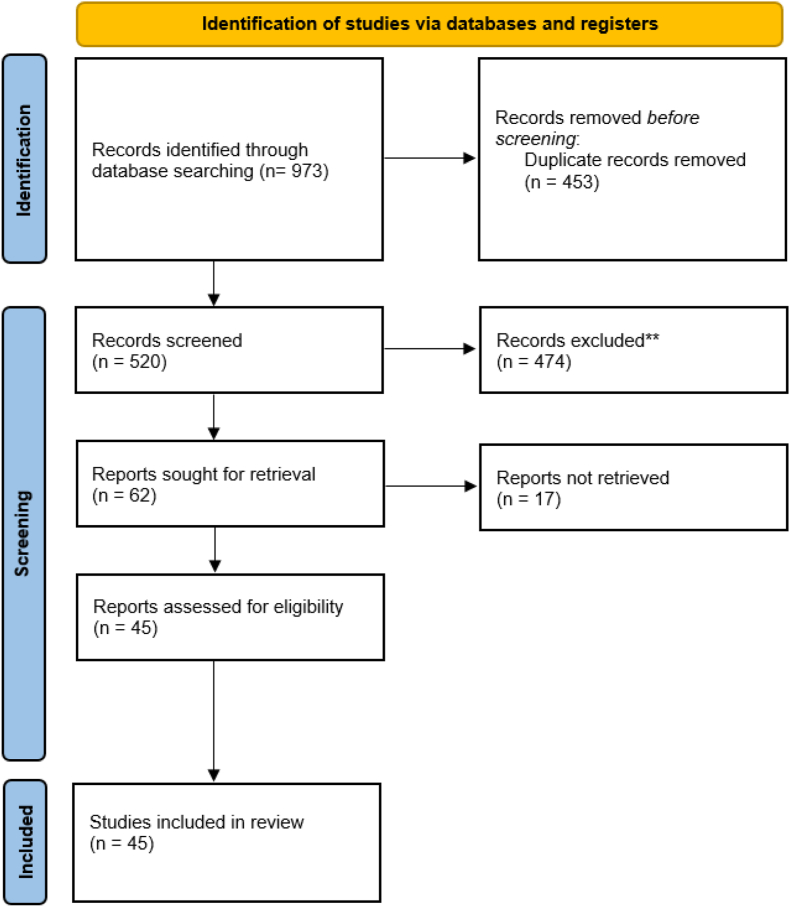
PRISMA flow chart.

The paper selection process is shown in Fig. 2. Main included article are shown in Table 1.

**Table 1:** Main selected papers.

First author	Year	Types of paper
Moe K.	2007	Case report
Moe KS	2010	Case report
Moe KS	2011	Case report
Chen HI	2015	Case report
Alqahtani A	2015	Anatomical investigation
Chen	2015	Case report
Tham T	2015	Case report
Dallan I	2015	Case report
Alqahtani A	2015	Anatomical investigation
Locatelli D	2016	Literature review
Ramakrishna R	2016	Case report
Dallan I	2017	Anatomical investigation
Almeida JP	2017	Anatomical investigation
Di Somma A	2018	Anatomical investigation
Jeon C	2018	Case report
Zoia C	2018	Case report
Lin BJ	2019	Anatomical investigation
Gerges	2019	Anatomical investigation
Luzzi S	2019	Literature review
Miller	2019	Case report
Lubbe D	2019	Case report
Zoli M	2020	Literature review
Miller	2020	Case report
Park HH	2020	Case report
Kong	2020	Expert opinion
Mahmoud	2021	Case report
Lim	2021	Anatomical investigation
Houlihan	2021	Literature review
Agosti	2021	Anatomical investigation
Vural A	2021	Literature review
Houlihan LM	2021	Literature review
Corrivetti F	2022	Anatomical investigation
Di Somma A	2022	Expert opinion
Corvino S	2022	Literature review
De Rosa A	2022	Anatomical investigation
DI Somma A	2022	Literature review
Zoia C	2022	Expert opinion
Guizzardi G	2022	Expert opinion
Schwartz TH	2022	Case report
Dallan I	2022	Expert opinion
De Rosa	2022	Anatomical investigation
Ben Cnaan R	2022	Case report
Han	2023	Case report
Di Somma	2023	Literature review
Zoli M	2023	Case report

## 4 Discussion

### 4.1 Trans-nasal endoscopic approach to the orbit

At the end of the 1980s, transnasal orbital endoscopy (TNOE) was initially applied for dacryocystorhinostomy (McDonogh e and Meiring, 1989) and orbital decompression for Grave's disease (Kennedy et al., 1990).

### 4.2 Indications

A fundamental principle reigns over the choice of TNOE for orbital access: the surgical corridor should not cross the optic nerve (ON) to avoid direct manipulation and subsequent deficit. Based on that principle, lesions best suitable for TNOE are located medially and inferiorly to the ON. Nowadays, the main indications for TNOE are orbital and optic canal decompression (Grave's disease, traumatic optic neuropathy), medial orbital wall fracture repair, and medial extra/intraconal orbital apex lesions.

Orbital cavernous hemangioma is the most common benign orbital tumor and the third most common orbital mass lesion (Calandriello et al., 2017). Recently, a consensus panel endorsed a Cavernous Hemangioma Exclusively Endonasal Resection (CHEER) staging system (El Rassi et al., 2019) in order to standardized TNOE approach to this pathology. Features to be considered are the anatomic relations between the tumor and adjacent structures such as the ON, medial rectus muscle (MRM) and inferior-medial trunk of the ophthalmic artery (IMT).

### 4.3 Relevant surgical anatomy

To access the medial orbital compartment, a standard endoscopic sphenoidotomy is initially performed, with further ethmoidectomy and maxillary sinus opening. Once the lamina papyracea is exposed, it is possible to enlarge the sphenoidotomy to visualize the ON bony bulging. Opening of the lamina papyracea, representing most of the medial orbital wall, should be done below the level of the ethmoidal foramina to avoid injury to the ethmoidal arteries (Castelnuovo et al., 2015). Finally, the periorbita is exposed and sharply opened to access the extraconal medial compartment.

Extraconal space is mainly filled with fat and connective septa, both less evident near the orbital apex. After removing the fat by dissection, the medial muscular wall, composed of the MRM and the inferior rectus muscle (IRM), is exposed. Usually, the safest access to the intraconal compartment is gained between the following two: the anterior ethmoidal artery usually passing between the MRM and the superior oblique muscle (SOM) and the posterior ethmoidal artery passing above the SOM (Castelnuovo et al., 2015). The neurovascular structures of the intraconal space are kept in place between fatty lobules divided by fibrous septa.

**Fig. 3:**
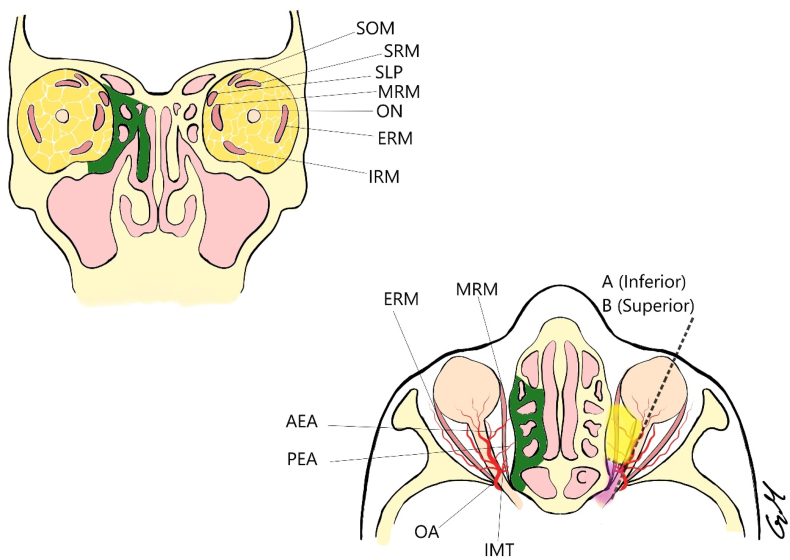
Transnasal endoscopic approach. AEA Anterior Etmoidal Artery, ERM External Rectus Muscle, IMT Inferior Medial Trunk, MRM Medial Rectus Muscle, OA Ophtalmic artery, ON Optic Nerve, PEA Posterior Etmoidal Artery, SOM Superior Oblique Muscle, SLP Superior Levator Palpebrae, SRM Superior Rectus Muscle.

Conceptually, the intraconal space from the TNOE perspective could be divided into three different zones of surgical complexity, based on the course of the IMT. Zones A and B are between the globe (anteriorly) and the IMT (posteriorly) and are safer to dissect. Zone A is the least technically demanding one, inferior to the MRM and with the largest space for manipulation. Zone B comprises the area superior to the MRM with the ethmoidal vessels and the ophthalmic artery (OA). Zone C is posterior to the IMT, where the MRM lies near the ON, thus allowing just minimal handling to avoid neural damage (Bleier et al., 2014) (Fig. 3).

### 4.4 Muscle retraction

To manage the intraconal dissection, it is possible to retract the MRM and the IRM in various ways: externally by placing a vessel loop at the MRM insertion on the globe or detaching it from the globe and passing it medially from the orbit into the nasal cavity, to be reattached at the end of the surgery.

Endoscopic methods include transseptal retraction through a septal window, anchoring the MRM to the septum with a stitch, intranasal use of a vocal fold retractor or direct manual transseptal retraction with a blunt instrument (Reshef et al., 2021).

### 4.5 Reconstruction

The decision to restore the medial orbital wall remains controversial. An accurate reconstruction could prevent middle- and long-term complications such as enophthalmos or diplopia. On the other hand, it increases the risk of acute orbital compartment syndrome in case of post-operative bleed or swelling. Generally, it is recommended to drape orbital fat over the MRM to prevent future scarring and muscular retraction, resulting in limited ocular movements. Various techniques have been used to restore the medial orbital wall, both rigid (autologous bone, synthetic materials) and semi-rigid (nasoseptal flap variations) (Reshef et al., 2021).

### 4.6 Outcome

Several studies have assessed the mid-to long term clinical outcome of TNOE, suggesting its efficacy and safety. Symptoms such as diplopia, enophthalmos, and cerebrospinal fluid leaks are transient in most cases (around 70%) (Dubal et al., 2014). Notably, intraconal approaches have a higher incidence of complications, incomplete resection and need of reconstruction (Bleier et al., 2016).

### 4.7 Trans-orbital endoscopic approach to the orbit

Since the first case series of an endoscopic approach to the orbit by Norris and Cleasby in 1981 (Norris e and Cleasby, 1981) for foreign body removal and tumor biopsies, the field enormously expanded, varying indications, techniques, surgical routes and targets.

The modern concept of transorbital neuroendoscopic surgery (TONES) was first presented at the 91st Annual Meeting of the Pacific Coast Oto-Ophthalmological Society by Kris Moe in 2007 (Moe, 2007) and subsequently published in 2010 (Moe et al., 2010). From that time, the concept of the sino-orbito-cranial interface as a distinct region of endoscopic interest has evolved (Alqahtani et al., 2015).

As presented above, the orbit can be endoscopically approached with two main goals: to treat intraorbital pathology and to obtain a key-hole passage to intracranial compartments with minimally disruptive approach (Miller et al., 2020). In both cases, the co-planarity between the orbital axis and the endoscope, meaning that the endoscope longitudinal axis runs parallel to the orbital cavity axis, provides the main operative advantage regarding operative angles, parenchymal retraction and shortness of surgical trajectory. Eventually, a superior-lateral orbital rim osteotomy has been proposed to further increase the surgical field width (Lim et al., 2021).

### 4.8 Indications

Conceptually, the main indication to perform a transorbital approach is a deep-seated lateral lesion, superior or inferior to the ON, which would be difficult to reach from a transnasal perspective or require an extensive transcranial approach.

Indication for TONES are currently increasing, but include CSF leak (iatrogenic, congenital or traumatic) (Ramakrishna et al., 2016), trigeminal schwannoma (Park et al., 2020), spheno-orbital meningioma (Dallan et al., 2015; Di Somma et al., 2023; Kong et al., 2020), temporal glioma (Chen et al., 2015), esthesioneuroblastoma (Raza et al., 2013), intraorbital/epidural/frontal abscess (Ramakrishna et al., 2016), fibrous dysplasia (Tham et al., 2015), Paget disease (Raza et al., 2013), hemangioma (Dallan et al., 2015), paranasal sinus mucoceles (Miller et al., 2019), ligation of the maxillary artery (Mahmoud et al., 2021) and intraconal meningioma of the orbital apex (Luzzi et al., 2019). Alongside this expanding field of surgical indications, it is to say that the true benefit of the transorbital route for a pure intracranial pathology has still to be demonstrated and clear evidence of surgical and clinical superiority still lacks (Zoia et al., 2022; Vural et al., 2021).

### 4.9 Relevant surgical anatomy

For sure, a deep understanding of the anatomy of the eyelid is essential. The superior lid crease (SLC) approach is the most commonly employed and provides good visualization of the lateral superior orbital compartment, frontal sinus, supraorbital and posterior-central portions of the anterior cranial fossa and lateral part of the middle cranial fossa. Conversely, the precaruncular incision can approach medial and inferior lesions, exposing the lamina papyracea, ethmoidal arteries, cavernous sinus, parasellar and paraclinoid tracts of the internal carotid artery, ON. Alternatively, inferior lid crease (ILC) and lateral retrocanthal (LC) incisions are used to address inferiorly located pathologies (Fig. 1).

The dissection of orbicularis muscles should be done following the direction of the fibers. The intraorbital dissection must occur in a plane between the periosteum and the periorbital connective tissue (Guizzardi et al., 2022). For intraorbital pathologies, after the incision of the periorbita to reach the extraconal compartment, dissection and removal of the lesion follow the same anatomical principles detailed for the transnasal approach.

For intracranial pathologies, several studies investigated the potential anatomical exposure of the anterior and middle cranial fossa after drilling the osseous borders (Guizzardi et al., 2022; Di Somma et al., 2018; Matano et al., 2022). Several bony landmarks are used in literature to perform the craniectomy: the most common used is the Superior Orbital Fissure (SOF), followed by the Sagittal Crest (Corrivetti et al., 2022), the Inferior Orbital Fissure (IOF) and the Great Sphenoid Wing (GSW) (Vural et al., 2021). Recently, anatomical exploration has expanded, and even the tentorial area (De Rosa et al., 2022), the infratemporal fossa (Gerges et al., 2019) and the hippocampus (Chen et al., 2015) have been investigated as one of the potential targets of the transorbital approach.

### 4.10 Reconstruction

For most procedures limited to the orbit or with small craniotomies, detailed reconstruction is not necessary (Almeida et al., 2017, Dallan et al., 2017). The orbital contents act as a self-sealant keeping them in place within the structures. For larger craniotomies or involvement of sinonasal spaces, a watertight closure and reconstruction are mandatory to avoid infections and CSF leakage. A multilayer technique is usually employed, both with autologous or synthetic grafts, following the principles of transnasal endoscopic reconstruction (Chen et al., 2015), (Lubbe et al., 2020, Jeon et al., 2018) even if a standardized closing technique is, however, not yet established. When the lateral orbital rim is drilled or completely removed for extended approach, reconstruction can be achieved with a miniplate or titanium mesh (Lin et al., 2019) in order to prevent post-operative enophthalmos.

### 4.11 Outcome

Known complications of transorbital approach are proptosis, diplopia, V2 numbness (6%, 5%, and 6%, respectively), meningitis, surgical site infections, CSF leak, levator muscle dysfunction, epiphora, orbital pseudomeningoceles (Ramakrishna et ai., 2016, Park et al., 2020, Chen et al., 2015, Tham et al., 2015, Moe et al., 2011). Notably, the most common sequelae are transient and stable post-operative deficits are very rare in literature. The mean intraoperative blood loss is reported between 60 mL (for orbital tumors) to 103 mL (for cavernous sinus and Meckel's cave lesions) (Han et al., 2023). A recent systematic literature review found that transorbital intervention is overall associated with notable neurological improvement of deficits such as extra-ocular movements and visual acuity (Houlihan et al., 2021b). The SLC approach is associated with the highest rate of complications, but it is unclear if it depends from the fact that it is the most commonly performed incision.

### 4.12 Future perspective

Transorbital surgery has evolved from the necessity to find alternative access to certain orbital tumors and achieve better results in terms of exposure and, at the same time, to maintain a functional-aesthetic balance. Through the decades, different specialties developed proper methods to access this shared area of interest. Endoscopic approach to the orbit is known since 1981, but it remained a matter of few isolated case reports, almost unknown. During the last years a renewed interest brought to the first quantitative studies on the subject and an extensive pre-clinical anatomical investigation on the possibilities of much more complex surgeries through this access.

We are now at a new stage of transorbital surgery, and a new paradigm is emerging. The conceptual frameshift regards seeing the orbit as a target that can be accessed simultaneously from different perspectives, depending on the surgical goals and patient-specific characteristics. Also, it is important to underlined the necessity of a multidisciplinary approach to this kind of surgery, involving ophthalmologist, maxillo-facial surgeron, ENT surgeon and neurosurgeon to provide the best possible patient-tailored treatment and follow up. The idea of the “multiportal” orbital surgery (Alqahtani et al., 2015), (Dallan et al., 2015), (Castelnuovo et al., 2013), (Lubbe et al., 2019), transnasal, transorbital and transcranial, already has some supporting data: the combined trans-orbital and transnasal approach is associated with less post-operative diplopia and V2 numbness compared to uniportal (transorbital or transnasal) access (Dallan et al., 2015) with adequate corridor to several skull base tumors (Di Somma et al., 2022a). It is essential to develop a detailed and evidence-based (Agosti et al., 2021) knowledge of advantages and limitations of every available approach, to avoid surgical excesses based on the unavoidable fashion phenomena.

Another aspect of the topic that needs to be addressed interdisciplinary, is clinical research. To date, papers regarding transorbital surgery are continuously published with an increasing trend, but, at the same time, the first specific systematic review failed to complete a metanalysis because of the paucity of data (Houlihan et al., 2021b), and it is stated that “The dialogue on technical and operative superiority is premature” (Houlihan et al., 2021b). Homogeneous nomenclature of approaches and anatomical landmarks, consistency across studies in reporting outcomes, broad shared classifications and scores, and well-designed randomized clinical trials are strongly advocated to produce high-level scientific evidence and further standardize procedures and results.

Still, transorbital surgery is currently performed in a few highly specialized centers, limiting the possibility of spreading skills and knowledge among new-generation surgeons. In this proposal, Dallan et al. proposed that to gain confidence with endoscopic transorbital procedures, at least 50 cases are required, with at least 20 cases/year to maintain it (Dallan et al., 2022). As with every other technique, also trans-orbital surgery has different levels of complexity (Di Somma et al., 2022b), and an adequate training program should progressively encompass all of it. Meanwhile, new methods and technique for neurosurgical training are developing and found initial scientific validation (Choudhury et al., 2013, Clark et al. 2017, Nicolosi et al., 2021, Paro et al., 2022, Petrone et al., 2022, Lee et al., 2022). Perhaps, in the following years, an approach to the actual patient in a classic surgical room scenario will be the last step of training involving hands-on courses (Stienen et al., 2016, Moiraghi et al., 2020), augmented reality (Davidovic et al., 2021, Haemmerli et al., 2021), artificial intelligence and real-life simulators (Stud et al., 2018, Meling and Meling 2021, Perin et al, 2022, Perin et al., 2021). Specific and dedicated applications of these principles are needed for transorbital endoscopic surgery to incorporate this topic into the common neurosurgical knowledge fully.

In the years to come, collaboration will emerge as the crucial factor in fostering the advancement of endoscopic orbital surgery across Europe and in establishing a robust, harmonious, and interdisciplinary approach to the subject.

Raising awareness of this topic among the general public is of utmost importance. Organizing educational events such as courses, webinars, or similar initiatives focused on orbital endoscopy will engage the community and enable surgeons to enhance their clinical proficiency in this methodology. Early mastery of the intricate neuroanatomical aspects of the orbit is imperative.

Partnering with other scientific societies (ENT, Maxillofacial Surgery, Ophthalmology) will amalgamate evidence and formulate comprehensive guidelines. While this paper represents the neurosurgeon's perspective, it necessitates integration with diverse surgical and clinical insights. An adept approach would encompass multiple viewpoints, culminating in a comprehensive interdisciplinary guideline to inform decisions in orbital surgery.

Similar to all rare medical conditions, establishing a case registry forms the cornerstone for addressing current clinical queries. A survey encompassing the aforementioned specialties will ascertain prevailing clinical practices, individual patient caseloads, and available training resources. Creating a directory of active centers can spotlight potential institutions for subspecialty training, fostering collaborations and facilitating professional mobility among colleagues who aspire to specialize in orbital surgery.

## 5 Conclusion

In conclusion, the orbit, its boundaries, its contents and its approaches are a borderland, continuously evolving through the interactions of different surgeons. Also, it has been like this since the first attempt to move away from the classical destroying approach, the orbital exenteration. Endoscopic navigation has the potential to provide useful access to different intracranial compartments, previously reachable only via an extensive tissue dissection. The following years will be crucial to further expand and precise indications, techniques, outcomes and complications through data obtained from high-level scientific studies. Extending this knowledge to the next generations will require the evolution of dedicated and multidisciplinary training programs so that the transorbital, transnasal and combined approaches becomes commonly available to patients by the orbital surgeons of tomorrow.

## Funding

The authors did not receive support from any organization for the submitted work.

## CRediT authorship contribution statement

**C. Zoia:** Conceptualization, Methodology, Writing – review & editing. **G. Mantovani:** Formal analysis, and, Investigation, Writing – original draft, Writing – review & editing. **M. Müther:** Conceptualization, Methodology, Writing – review & editing. **E. Suero Molina:** Writing – review & editing. **A. Scerrati:** Writing – review & editing. **P. De Bonis:** Writing – review & editing. **J.F. Cornelius:** Writing – review & editing. **P.H. Roche:** Writing – review & editing. **M. Tatagiba:** Writing – review & editing. **E. Jouanneau:** Writing – review & editing. **R. Manet:** Writing – review & editing. **H.W.S. Schroeder:** Writing – review & editing. **L.M. Cavallo:** Writing – review & editing. **E.M. Kasper:** Writing – review & editing. **T.R. Meling:** Writing – review & editing. **D. Mazzatenta:** Writing – review & editing. **R.T. Daniel:** Writing – review & editing. **M. Messerer:** Writing – review & editing. **M. Visocchi:** Writing – review & editing. **S. Froelich:** Writing – review & editing. **M. Bruneau:** Writing – review & editing. **G. Spena:** Writing – review & editing, Supervision.

## Conflict of interest

The authors have no competing interests to declare that are relevant to the content of this article.
